# Effectiveness of Mobiderm® bandages in the treatment of cancer-related secondary lymphedema: A pilot study

**DOI:** 10.1097/MD.0000000000030198

**Published:** 2022-09-02

**Authors:** Sung Cheol Cho, Sang Gyu Kwak, Hee Kyung Cho

**Affiliations:** a Department of Physical Medicine and Rehabilitation, Catholic University of Daegu School of Medicine, Republic of Korea; b Department of Medical Statistics, Catholic University of Daegu School of Medicine, Republic of Korea.

**Keywords:** complex decongestive therapy, compression bandaging, maximal circumference difference, Mobiderm® bandages, secondary lymphedema

## Abstract

Secondary lymphedema is a clinically incurable disease that commonly occurs following surgical cancer treatment and/or radiation. One of the most common forms of lymphedema treatment is complete decongestive therapy (CDT). This study aimed to investigate the clinical effects of new compression bandages (Mobiderm® bandages) in patients with secondary lymphedema after cancer treatment. This study included 17 patients with ipsilateral limb lymphedema after cancer treatment (one male and 16 female patients; age, 45–80 years). Patients were divided into the Mobiderm® bandage group (n = 9) and classical bandage group (n = 8). The International Society of Lymphology (ISL) stage was also evaluated. Limb circumference was measured at 5 to 6 sites per limb to identify the maximal circumference difference (MCD) between the affected and unaffected limbs. Pre-and posttreatment MCD were analyzed. After intensive CDT, both the Mobiderm® bandage group (1.2 ± 0.56 cm) and classical bandage group (0.85 ± 0.40 cm) had a significant decrease in MCD compared to pretreatment (*P* < .05). However, in patients with ISL stage 2, the mean MCD decrease rate was greater in the Mobiderm® bandage group (22.82 ± 10.92 %) than in the classical bandage group (12.18 ± 8.1 1%)(*P* = .045). Both new bandages (Mobiderm® bandages and classical bandages) reduced the circumference of limb edema in patients with secondary lymphedema after cancer treatment. This study findings suggest that Mobiderm® bandages as an alternative modality for controlling ISL stage 2 lymphedema.

## 1. Introduction

Lymphedema is defined as a medical condition in which protein-rich fluid accumulates in cutaneous and subcutaneous tissues because of a dysfunction in lymphatic drainage.^[1,2]^Secondary lymphedema arises after resection or obstruction of the lymphatic system as a consequence of lymph node dissection and radiotherapy for cancer.^[3,4]^ Chronic lymphedema leads to inflammation, adipose tissue hypertrophy, and fibrosis.^[2]^ As edema progresses, the disease can cause functional, physical, and psychological defects that can affect a patient’s quality of life.^[5,6]^ One of the most common forms of treatment for lymphedema is complete decongestive therapy (CDT), which consists of 2 stages. The first stage, also known as the intensive phase, includes manual lymphatic drainage, compression bandaging, self-exercise, and skin care. The second phase, also known as the self-management phase, consists of self-administered lymphatic drainage, daily application of compression bandaging, and self-exercise.^[7,8]^ In particular, compression bandaging has been suggested to be responsible for the most significant reduction of the limb swelling.^[9]^ Compression bandaging increases interstitial tissue fluid pressure and lymph node uptake. When the muscles contract against the compression bandages, they contract against a high working pressure and this enhances lymphatic and venous flow, resulting in the reduction of edema.^[10]^ To improve self-care and prevent rebound swelling, multilayered compression bandaging techniques have generally been used. Multilayered compression bandaging involves the uses of bandages with varying degrees of compression, additional materials, and supportive materials. It usually consists of stockinettes, gauze bandages, padding materials, and low-stretch bandages. Tubular bandages absorb sweat and protect the skin from subsequent layers. Foams form a “padding” layer of multilayered compression bandaging that helps to cushion the limbs and bony areas, prevent skin irritation, and evenly distribute the gradient pressure created by low-stretch bandages.^[11]^ Low-stretch bandages are less likely to impinge on skin folds and provide a semirigid support structure for the muscles to contract against, which enhances lymph and venous flows, and helps to reduce edema.^[10]^ Recently, several manufacturers have designed various compression-bandage materials.

One of them is the Mobiderm® bandages, which is designed and manufactured by the French company, Thuasne. It is a medical bandage that consists of padded foam blocks encased between 2 nonwoven bandages. Its structure is made up of many small capsules of compressible material, which creates compression gradients between adjoining areas, as well as compression/decompression effects on application. This induces the displacement of edema from higher-pressure zones to the lower-pressure zone, and also acts on venular reabsorption. However, little is known about the efficacy of Mobiderm® bandages in the treatment of lymphedema. This study aimed to compare the efficacy of Mobiderm® bandages to that of classical compression bandages.

## 2. Patients and methods

### 2.1. Patients

**Figure 1.**
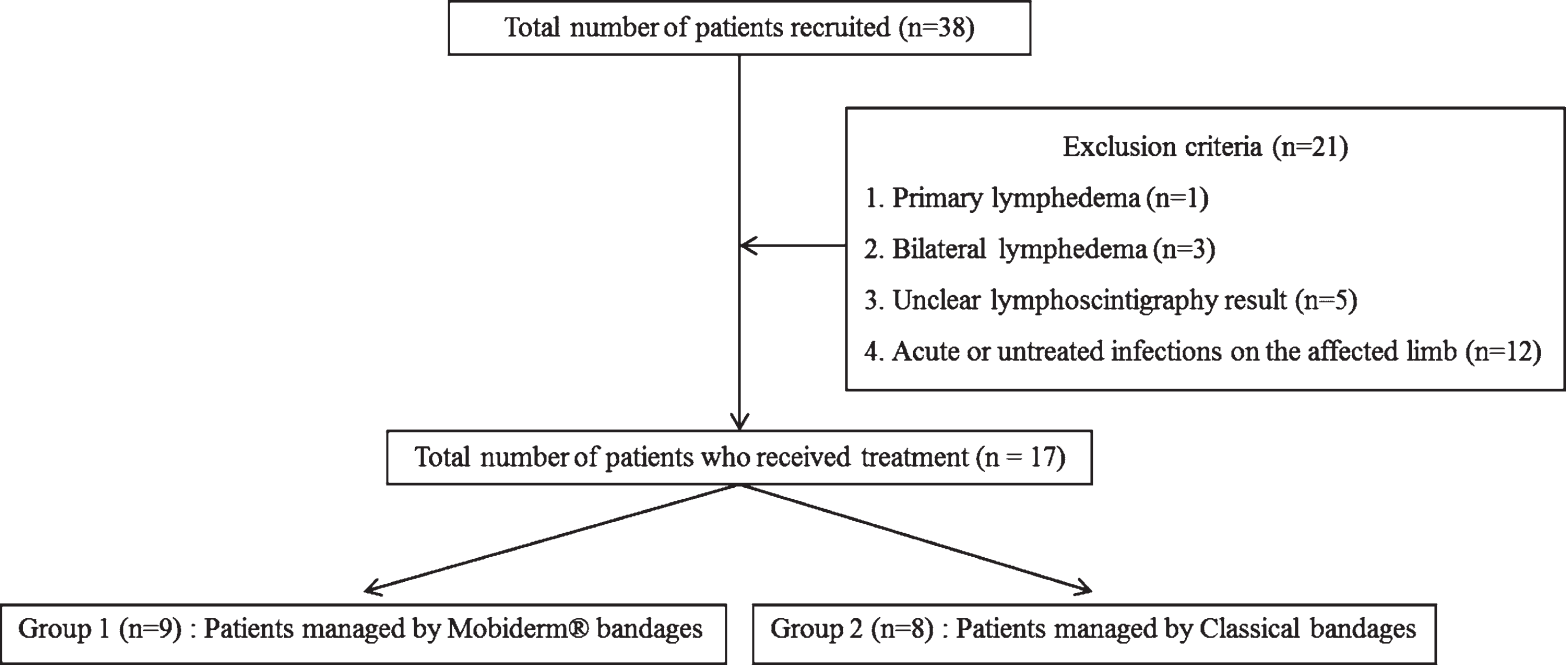
Flow chart of this study.

A retrospective medical chart review was performed between May 2017 and December 2019, and included 38 patients who were admitted to the Department of Physical Medicine and Rehabilitation for the treatment of secondary lymphedema. The inclusion criteria were as follows: (a) patients who were clinically diagnosed with unilateral secondary lymphedema due to cancer treatment; (b) confirmed diagnosis of lymphedema by lymphoscintigraphy; and (c) cases in which CDT was performed daily for 7–14 days. The exclusion criteria were as follows: (a) primary lymphedema; (b) bilateral lymphedema; (c) unclear lymphoscintigraphy results; and (d) patients with acute or untreated infections on the affected limb. A total of 17 subjects who satisfied the inclusion and exclusion criteria were allocated and divided into 2 groups: group 1 (Mobiderm® bandage group) and group 2 (classical bandage group) (Fig. 1). The study protocol was approved by the Institutional Review Board of Daegu Catholic University Medical Center and was conducted in accordance with the principles of the Declaration of Helsinki. Given the retrospective nature of the study, obtaining informed consent was not required.

### 2.2. Compression bandaging

**Figure 2.**
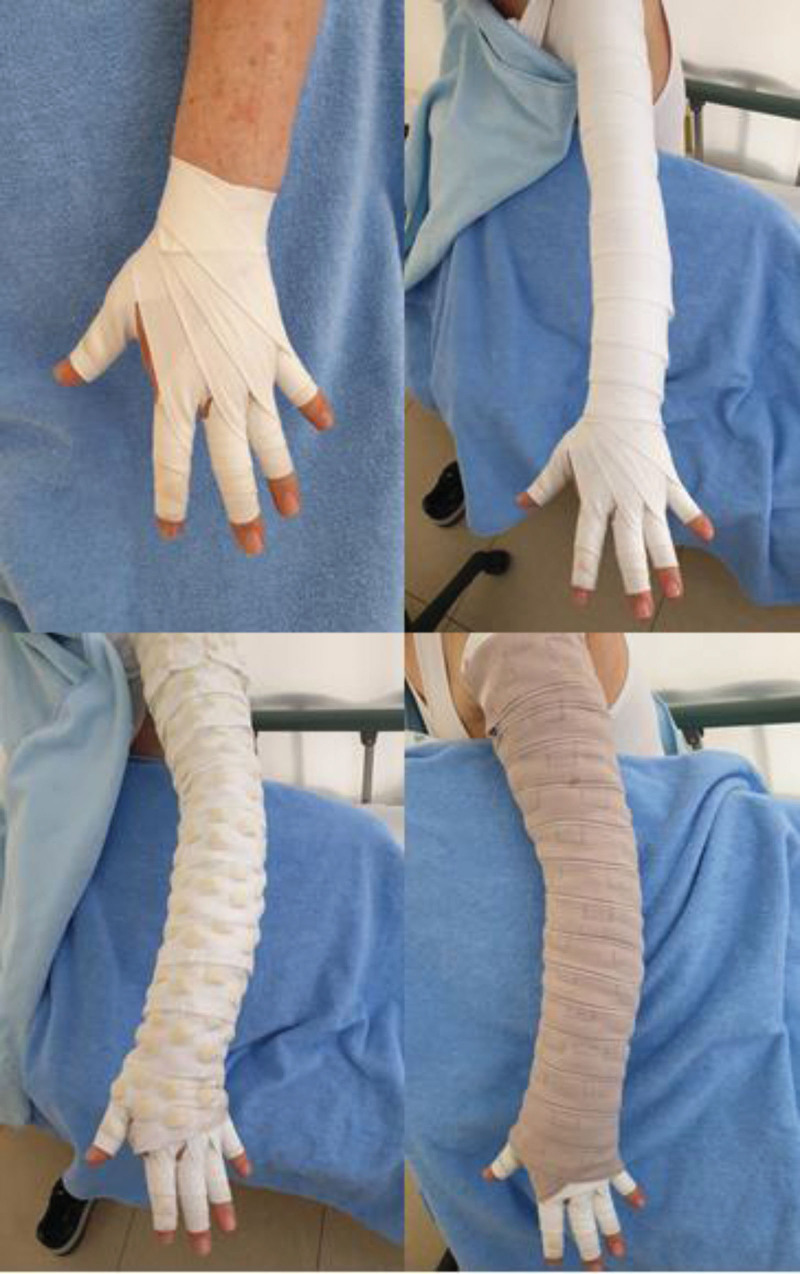
Application of Mobiderm® bandages in the upper limb.

Multilayered compression bandaging was performed using the following method. First, a stockinette (tubular bandage) was applied over the lymphedematous limb. Second, a gauze bandage area that was made from elastic cotton material was applied to the fingers and toes. Third, foam padding was applied on top of the stockinette under the low-stretch bandages. Fourth, low-stretch bandages were applied to the limbs, and adhesive tapes were used to hold the bandages in place.^[11]^ The patients in group 1 were treated with the application of foam padding with Mobiderm® bandages, which consisted of padded foam blocks encased between 2 nonwoven bandages (Fig. 2). The patients in Group 2 were treated with nonwoven synthetic padding bandages named Cellona (Lohmann & Rauscher). During the admission period, all patients underwent CDT, which consisted of manual lymphatic drainage by a qualified therapist, self-exercises to enhance lymphatic drainage, and daily skin care. All patients were instructed to apply compression bandaging for 21 to 23 h daily.

### 2.3. Lymphedema assessment

Before treatment, all patients underwent extensive physical examination of the edematous limb. The circumferences of the affected and unaffected limbs were measured using a nonelastic measuring tape before and after 7–14 days of treatment. The measurement points of the upper limb circumference were the elbow (mid-point between the medial and lateral epicondyle), 10 cm proximal to the elbow, 10 cm distal to the elbow, wrist joint (midpoint of the wrist crease), and the metacarpophalangeal joint. The measurement points of the lower limb circumference were the upper margin of the patellar, 10 cm and 20 cm proximal to the upper margin of the patellar, lower margin of the patellar, 10 cm and 20 cm distal to the lower margin of the patellar, and the ankle joint (the shortest distance between the medial malleolus and lateral malleolus). To ensure the reliability of the measurement, the circumference was measured twice by the same clinician, and the mean value was recorded. Among the measurement points, the maximal circumference difference (MCD) between the affected and unaffected limbs was recorded.^[12]^

### 2.4. Statistical analysis

The normality of each parameter was tested using the Shapiro–Wilk test. Data are presented as the mean values and standard deviations of the evaluated parameters. The Wilcoxon signed-rank test was used to evaluate the change in clinical data from baseline to posttreatment in both groups, and the Mann–Whitney U test was used to compare between the groups. Statistical significance was set at *P* < .05.

## 3. Results

### 3.1. Patients’ characteristics

Our study included 17 patients (one male and 16 female patients; mean age, 74.4 ± 10.3 years) with secondary lymphedema that was confirmed using lymphoscintigraphy after cancer treatment. According to the grading system from the International Society of Lymphology (ISL), 4 patients had stage 1 lymphedema, and 13 patients had stage 2 lymphedema. Regarding the patients’ clinical characteristics, no significant difference was found in the duration of lymphedema, severity of lymphedema, and having received surgery, chemotherapy and radiotherapy between the 2 groups; however the duration of cancer related operation was different between both groups (193.11 ± 113.09 months in the mobiderm® bandage group vs 69.69 ± 50.69 months in the classical bandage group) (*P* = .026) (Table 1).

**Table 1:** Baseline characteristics of participants and comparison between Mobiderm® bandage group and classical bandage group.

Variable	Group 1 (n = 9)	Group 2 (n = 8)	P-value
Age (yr)	63.00 ± 4.27	57.83 ± 3.86	0.283
Gender (male: female)	9:0	7:1	0.687
Affected side (right: left)	6:3	4:4	0.853
BMI	23.44 ± 3.78	22.50 ± 4.04	0.774
Duration of lymphedema (mo)	12.56 ± 14.28	3.38 ± 2.71	0.349
Duration of cancer relatedsurgery (mo)	193.11 ± 113.09	69.69 ± 50.69	0.022*
ISL stage (1:2)	2:7	2:6	0.586
Surgery (yes: no)	9:0	7:1	0.486
Chemotherapy (yes: no)	4:5	5:3	0.399
Radiotherapy (yes: no)	4:5	6:2	0.218

Values are presented as mean ± standard deviation or number.

Group 1 = Mobiderm® bandage group, Group 2 = Classical bandage group, BMI = body mass index, ISL = International society of lymphology.

P values represent between-group comparisons.

* *P* < .05.

### 3.2. Maximal circumference differences

**Figure 3.**
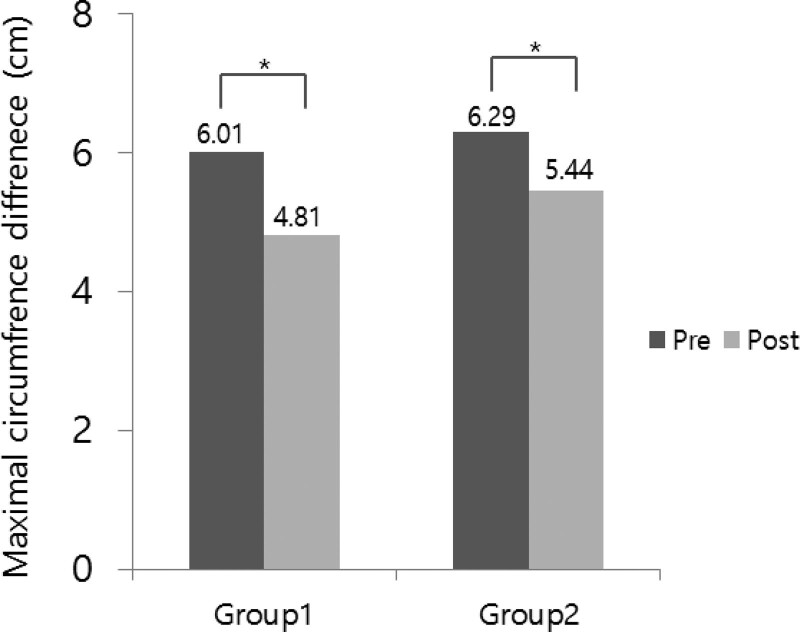
The maximal circumference difference (MCD) between affected and unaffected limb in Mobiderm® bandage group (Group 1) and classical bandage group (Group 2) at pretreatment (Pre) and after 7–14 days of complete decongestive therapy (Post). Values are presented as mean. *P < .05.

There was a significant difference in the MCD between the Mobiderm® bandage group and the classical bandage group at pretreatment and after 7 to 14 days of CDT (Fig. 3). The degree of the MCD difference before and after treatment was 1.20 ± 0.56 cm in the Mobiderm® bandage group and 0.85 ± 0.40 cm in the classical bandage group. The decrease rate of the MCD was 21.75 ± 9.72 % in the Mobiderm® bandage group and 16.01 ± 9.95 % in the classical bandage group, respectively, which was not statistically significant between the groups (*P* > .05). However, in patients with ISL stage 2 lymphedema, the decrease rate of the MCD before and after treatment was 22.82 ± 10.92 % in the Mobiderm® bandage group and 12.18 ± 8.11 % in the classical bandage group, which was statistically significant (*P* < .05) (Table 2).

**Table 2:** Degree of the maximal circumference difference (MCD) decrease and rate of the MCD decrease between pretreatment and after 7–14 days of complete decongestive therapy.

	Total (n = 17)		ISL stage 2 (n = 13)	
	Group1 n = 9	Group2 n = 8	Group1 n = 7	Group 2 n = 6
MCD decrease (cm)	1.20 ± 0.56	0.85 ± 0.40	1.31 ± 0.58	0.85 ± 0.42
MCD decrease rate (%)	21.75 ± 9.72	16.01 ± 9.95	22.82 ± 10.92*	12.18 ± 8.11

Values are presented as mean ± standard deviation or number.

Group 1 = Mobiderm® bandage group, Group 2 = Classical bandage group, ISL = International society of lymphology, MCD = maximal circumference difference.

* *P* < .05.

### 3.3. Adverse effects

There were no serious complications during study period in both groups.

## 4. Discussion

This study assessed the efficacy of Mobiderm® bandages by measuring the circumferences of lymphedematous limbs in patients with cancer-related secondary lymphedema.

Lymphedema is a well-established complication of cancer treatment for which no curative treatment exists. It is a disabling condition that causes functional, physical, and psychological defects that can affect a patient’s quality of life.^[5,6]^ One of the most common forms of lymphedema treatment is CDT. Previous studies have reported that CDT is an effective therapy for lymphedema.^[13–15]^ In particular, compression bandaging is the essential part of CDT, which is the gold standard for treatment of secondary lymphedema.^[16]^ However, multilayered compression bandaging is not an easy technique, and it requires sophisticated techniques, so experienced physiotherapist are required at the beginning of the therapy.^[17]^ Previous studies reported that compression bandages could result in mild complications such as pain, discomfort, and tightness of the limb.^[18]^Also compression bandaging is avoided in patients with loss of skin integrity as it can lead to infection and aggravate lymphedema.^[19]^Therefore, several compression bandage materials have been manufactured to reduce discomfort and improve patients’ compliance recently.

This pilot study evaluated the effectiveness of Mobiderm® bandages as a treatment technique for the management of secondary lymphedema. The Mobiderm® bandaging system is a multilayered compression bandaging technique. The most significant difference between Mobiderm® bandages and other bandages is that they are composed of foam blocks encased in soft adherent webbing. All patients underwent inpatient treatment with CDT including compression bandaging, manual lymphatic drainage, and exercise. Except for the type of bandages, both groups underwent the same treatment. All patients were evaluated using the ISL classification to determine the severity of secondary lymphedema. According to the ISL classification, lymphedema is divided into 4 stages (stage 0, 1, 2, and 3).^[20]^ Early-stage lymphedema is defined as stages 0 and 1, wherein subcutaneous fibrosis has not developed. In contrast, lymphedema at stage 2 or 3 is considered irreversible since subcutaneous fibrosis has already developed.^[20]^ In this study, the participants were patients with ISL stage 1 or 2 lymphedema. In the Mobiderm® bandage group, there were 3 patients with ISL stage 1, and 6 patients with ISL stage 2 lymphedema. In the classical bandage group, there were 2 patients with ISL stage 1 lymphedema and 6 patients with ISL stage 2 lymphedema. Our study showed a significant decrease of MCD in both the Mobiderm® (1.20 cm) and classical (0.85 cm) bandage groups after treatment. Moreover, we found that Mobiderm® bandages could be superior to classical bandages in terms of the level of decrease in limb circumference, especially in patients ISL stage 2 lymphedema. In the Mobiderm® bandages group, it was observed that the decrease in MCD was 1.31 cm and the decrease rate of MCD was 22.82 % after treatment in patients with ISL stage 2 lymphedema. In the classical bandage group, the decrease in MCD was 0.85 cm and the decrease rate of MCD was 12.18 % after treatment in patients with ISL stage 2 lymphedema.

Some noticeable differences were observed between the demographic features and characteristics of lymphedema in the 2 groups. The Mobiderm® bandage group had a longer duration of cancer-related surgery before CDT initiation. Due to the characteristics of tertiary hospitals, many patients visit the rehabilitation department after a long period of time without receiving any other treatment after lymphedema occurs. In the Mobiderm® bandage group, an average of 193 months had passed after cancer-related surgery, whereas in the classical bandage group, an average of 70 months had passed. The duration of lymphedema from onset showed a longer tendency in the Mobiderm® bandage group than in the classical bandage group (12.6 months vs 3.4 months), but this was not statistically significant.

The initial presentation of lymphedema is the accumulation of protein-rich interstitial fluid in the subcutaneous and subfascial tissues, resulting in limb swelling, heaviness, and pitting edema. Chronic lymph stasis typically stimulates an increase in the number of fibroblasts, adipocytes, and keratinocytes in the skin, in addition to promoting large infiltration of neutrophils. These changes further decrease lymphatic function and promote disease progression.^[2,5,6]^ As a result, chronicity of lymphedema was suggested as a negative predictive factor of a patient’s response to CDT; thus, early intervention is an important step in lymphedema management.^[9,21]^ Although the Mobiderm® bandage group, consisted of patients with long-standing lymphedema after cancer related surgery who presented late at the Department of Physical Medicine and Rehabilitation, we still observed good responsive to 7–14 days of intensive treatment using Mobiderm® bandages, and this was especially noticeable in patients with ISL stage 2 lymphedema. It is suggested that multiple foam paddings, which are encased in the Mobiderm® bandages at regular intervals, created a remarkable pressure difference between the supporting zone and the surrounding area and improved lymphatic flow in the affected limb. The results of this study have potential clinical implications. Based on the results of this study, we can suggest Mobiderm® bandages to the patients with a longer period of pathological conditions and the ones in the comparatively more severe lymphedema stage: ISL stage 2.

This study has several limitations. First, the sample size was too small to be considered statistically significant. Second, there was a lack of objective measurement methods to evaluate limb size, as we measured only the differences between the circumference of the affected and that of the unaffected limbs. Third, the long-term effects of each bandages were not assessed. Therefore, the long-term effects of Mobiderm® bandages should be evaluated in future studies with longer follow-up periods. Fourth, we did not evaluate patients’ functional outcomes or quality of life. Finally, the generalizability of this result is limited, because the data were collected from a single institution.

## 5. Conclusions

In conclusion, based on our observations, applying Mobiderm® bandages appears to be a new promising treatment option that can be used to control limb circumference in patients with cancer-related lymphedema. Our findings suggest that Mobiderm® bandages can reduce limb circumference, especially in patients with ISL 2 stage lymphedema. Our results should be confirmed in future studies with larger sample sizes and longer follow-up periods.

## Author contributions

Conceptualization: Hee Kyung Cho.

Data curation: Sung Cheol Cho.

Formal analysis: Sang Gyu Kwak.

Investigation: Hee Kyung Cho, Sung Cheol Cho.

Writing—original draft: Sung Cheol Cho, Hee Kyung Cho.

Writing—review and editing: Hee Kyung Cho.
